# Poly(ethylmethacrylate-co-diethylaminoethyl acrylate) coating improves endothelial re-population, bio-mechanical and anti-thrombogenic properties of decellularized carotid arteries for blood vessel replacement

**DOI:** 10.1038/s41598-017-00294-6

**Published:** 2017-03-24

**Authors:** Elena López-Ruiz, Seshasailam Venkateswaran, Macarena Perán, Gema Jiménez, Salvatore Pernagallo, Juan J. Díaz-Mochón, Olga Tura-Ceide, Francisco Arrebola, Juan Melchor, Juan Soto, Guillermo Rus, Pedro J. Real, María Diaz-Ricart, Antonio Conde-González, Mark Bradley, Juan A. Marchal

**Affiliations:** 1 Department of Health Sciences, University of Jaén, Jaén, Spain; 2 Biopathology and Regenerative Medicine Institute (IBIMER), Centre for Biomedical Research, University of Granada, Granada, Spain; 3 School of Chemistry, EaStCHEM, University of Edinburgh, King’s Buildings, Edinburgh, UK; 4 Department of Human Anatomy and Embryology, Faculty of Medicine, University of Granada, Granada, Spain; 5 Biosanitary Research Institute of Granada (ibs.GRANADA), University Hospitals of Granada-University of Granada, Granada, Spain; 6 Pfizer-Universidad de Granada-Junta de Andalucía Centre for Genomics and Oncological Research (GENYO), Granada, Spain; 7 Department of Pulmonary Medicine, Hospital Clínic; Institut d’Investigacions Biomèdiques August Pi i Sunyer (IDIBAPS), University of Barcelona, Barcelona, Spain; 8 Biomedical Research Networking Center on Respiratory Diseases (CIBERES), Madrid, Spain; 9 Department of Histology, Faculty of Medicine, Institute of Neuroscience, Biomedical Research Centre, University of Granada, Granada, Spain; 10 Department of Structural Mechanics, University of Granada, Politécnico de Fuentenueva, Granada, Spain; 11 Department of Hemotherapy and Hemostasis, Hospital Clinic, Centre de Diagnostic Biomedic (CDB), Institute of Biomedical Research August Pi i Sunyer (IDIBAPS), Barcelona, Spain

## Abstract

Decellularized vascular scaffolds are promising materials for vessel replacements. However, despite the natural origin of decellularized vessels, issues such as biomechanical incompatibility, immunogenicity risks and the hazards of thrombus formation, still need to be addressed. In this study, we coated decellularized vessels obtained from porcine carotid arteries with poly (ethylmethacrylate-co-diethylaminoethylacrylate) (8g7) with the purpose of improving endothelial coverage and minimizing platelet attachment while enhancing the mechanical properties of the decellularized vascular scaffolds. The polymer facilitated binding of endothelial cells (ECs) with high affinity and also induced endothelial cell capillary tube formation. In addition, platelets showed reduced adhesion on the polymer under flow conditions. Moreover, the coating of the decellularized arteries improved biomechanical properties by increasing its tensile strength and load. In addition, after 5 days in culture, ECs seeded on the luminal surface of 8g7-coated decellularized arteries showed good regeneration of the endothelium. Overall, this study shows that polymer coating of decellularized vessels provides a new strategy to improve re-endothelialization of vascular grafts, maintaining or enhancing mechanical properties while reducing the risk of thrombogenesis. These results could have potential applications in improving tissue-engineered vascular grafts for cardiovascular therapies with small caliber vessels.

## Introduction

Cardiovascular diseases, including coronary artery and peripheral vascular disorders, affect a large amount of the population worldwide each year, and as such vascular grafts for by-pass procedures or replacement of damaged vessels are in high demand in vascular surgery. Currently, vascular grafts involve the use of autologous vessels or synthetic inserts. Despite autografts being the gold standard replacement option with long-term patency rates, their availability is limited due to pre-existing damage or their use in previous procedures and the requirement of an additional clinical procedure. Alternative sources for vascular grafts are synthetic grafts made of Dacron^®^ and expanded polytetrafluoroethylene^1^. Synthetic grafts have been used successfully for large caliber vessel reconstructions; however, they are not suitable for the replacement of vessels smaller than 6 mm in diameter mainly due to the risk of thrombus formation and neointimal hyperplasia which could lead to lumen occlusion^1, 2^.

To address these limitations, novel tissue engineering strategies have emerged to develop alternative conduits for clinical applications^3^. Biocompatible vascular scaffolds are designed to resemble native tissue as well as their functional and biomechanical properties. In this sense, decellularization of naturally available biomaterials has been identified as a promising approach for vascular replacement of small-diameter vascular vessels^4^. Tissue decellularization consists of removing all cellular components leaving behind the extracellular matrix (ECM) along with the main structural components of a blood vessel, such as collagen and elastin, which will then act as a natural scaffold with mechanical properties close to those of native tissue^4^. Moreover, decellularized matrices are less immunogenic than native tissue in as much as most of the antigenic cellular components are removed resulting in more favorable scaffolds for transplantations^5^.

Consequently, decellularized allogeneic and xenogeneic ECM scaffolds are now being investigated for the reconstruction of a wide range of specialized tissues with potential therapeutic applications including their use for cardiovascular conduits^6–8^. However, some studies have shown thrombus formation after acellular graft implantation partly attributable to the lack of a viable vascular endothelium^9, 10^. In fact, when a vascular lesion is produced, platelets migrate to the affected area causing thrombus formation^11^. Moreover, the decellularization process leads to the exposition of highly thrombogenic collagen on the vascular wall and platelet adhesion. Thus, strategies to enhance adhesion of endothelial cells (ECs) or endothelial progenitor cells (EPCs) improving the rapid endothelialization of vascular scaffolds while reducing platelet adhesion are key for vascular tissue engineering^6, 12^.

Poly(ethylmethacrylate-co-diethylaminoethylacrylate) (8g7) is a biocompatible polymer that has been described to enhance viability, attachment and function of progenitor and mature ECs. Moreover, the coating of intra-vascular devices with this biopolymer has been shown to facilitate the attachment of ECs while minimizing platelet binding^13^.

In the present study, we demonstrated better endothelial coverage, reduced risk of thrombosis and comparable biomechanical properties of 8g7-coated decellularized arteries compared to native ones. The adhesion and growth of human umbilical vein endothelial cells (HUVECs) on the uncoated and polymer-coated luminal surface of acellular carotid arteries was examined through a variety of techniques, including scanning electron microscopy (SEM) and transmission electron microscopy (TEM). Reduced platelet aggregation to the polymer was established under physiological shear flow through perfusion of whole blood in a parallel-plate flow chamber. In addition, the biomechanics of native acellular and polymer coated decellularized arteries were compared with the native arteries. This study highlights the potential of combining synthetic polymer coatings with natural acellular vessels to develop novel tissue-engineered grafts for vascular repair.

## Results

### Capillary-like tube formation assay on biopolymer coated surfaces

**Figure 1:**
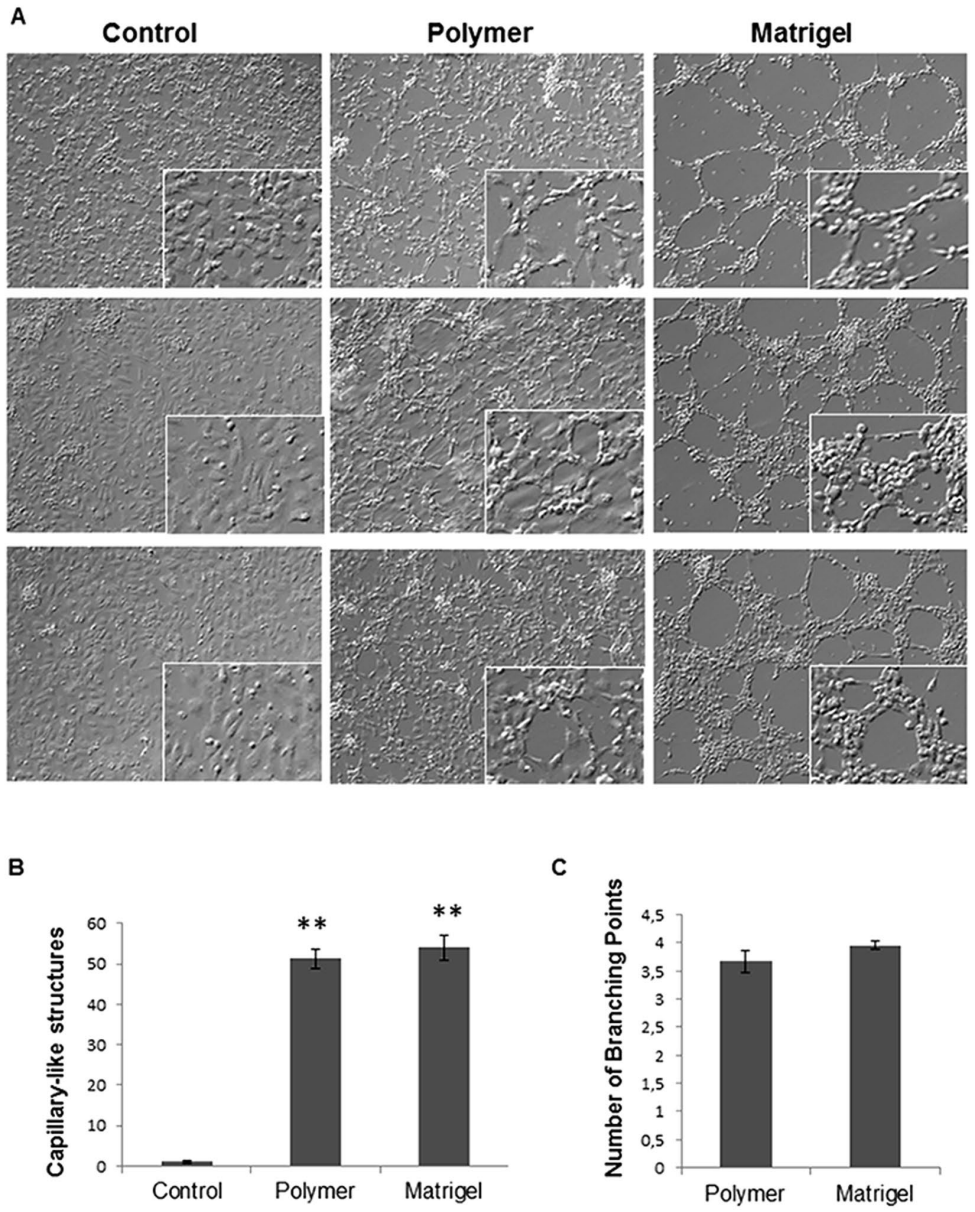
Capillary network formation assay after 4 h of culture. (**A**) Phase contrast microscopy of HUVECs grown for 4 hours on different surfaces: uncoated glass coverslips (control), polymer 8g7 coated coverslips and Matrigel (magnification; x10), inset shows higher magnification of the cells (magnification; x20). (**B**) Number of capillary-like structures measured. (**C**) Number of branching points per each capillary-like structure. Data from 3 independent experiments performed in duplicate are expressed as mean ± SD (**P < 0.05 vs. Control).

The tube formation assay was used to investigate the capacity of 8g7 polymer to induce Angiogenesis *in vitro* ^14^. HUVECs were seeded onto 8g7 polymer-coated glass coverslips as well as onto uncoated glass coverslips and 12-well plate coated with matrigel (Fig. 1). HUVECs were able to attach and grow on all surfaces (Fig. 1A). After 4 h, HUVECs cultured on both 8g7 polymer and matrigel formed capillary-like tubular structures, while uncoated glass coverslips did not (Fig. 1A). The number of capillary-like structures formed was counted at different areas of surfaces. No significant differences were found between number of capillary-like tubes formed by cells grown onto, 8g7 polymer or matrigel (Fig. 1B), indicating that HUVECs maintain their functional ability on both. The average number of cells that form a branch of the capillary-like structure when cells grown on polymer coated coverslips was 8 ± 0.29 with a mean number of branching points of 3.6 while an a average of 12 ± 0.3 cells were found to form tubes when cells were grown on matrigel with a mean number of branching points of 3.9 (P = 0.068) (Fig. 1C). Furthermore, the viability of HUVECs cultured onto polymer coated coverslips was not compromised as no dead cells were observed (data not shown).

### Platelet adhesion test on polymer-coated coverslips

**Figure 2:**
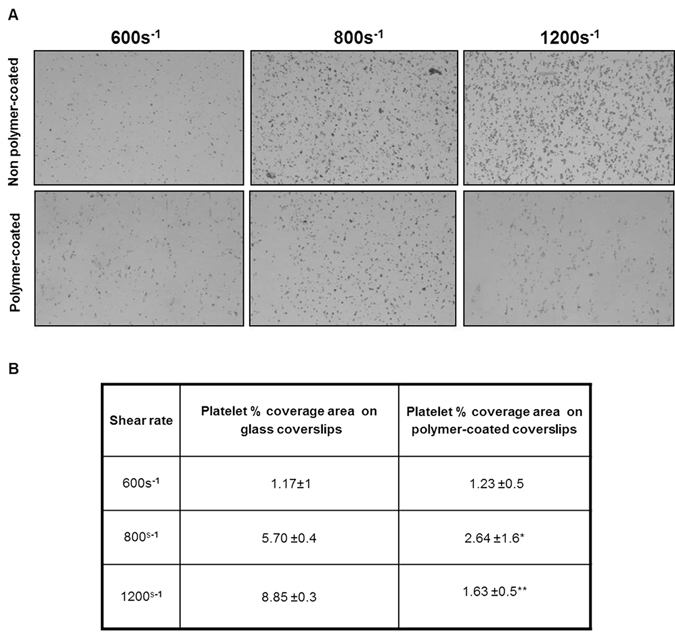
Platelet surface coverage on non polymer-coated and polymer-coated coverslips. Citrated blood samples were perfused through a parallel-plate perfusion chamber containing uncoated and polymer-coated coverslips at a shear rate of 600 s^−1^, 800 s^−1^ and 1200 s^−1^. (**A**) Representative images of phase contrast microscopy showing platelet surface coverage (magnification; x10). (**B**) Percentage of platelet surface coverage area under the different flow conditions. Data from 2 independent experiments performed in triplicate are expressed as mean ± SD (*P < 0.05), (**p < 0.01).

To determine whether platelets adhere onto the 8g7 polymer under flow conditions, citrated whole blood was perfused over polymer-coated and uncoated coverslips in a parallel plate chamber. The percentage of surface covered with platelets was determined following a shear rate of 600 s^−1^, 800 s^−1^ and 1200 s^−1^ for 4 min (Fig. 2A). Whilst no differences were seen under moderately low shear stress conditions (600 s^−1^) between uncoated and 8g7 polymer-coated coverslips, a significantly reduced number of platelets adhered to 8g7 polymer-coated coverslips under conditions of increased shear rate (800 s^−1^ and 1200 s^−1^) (Fig. 2B).

### Characterization of decellularized arteries

**Figure 3:**
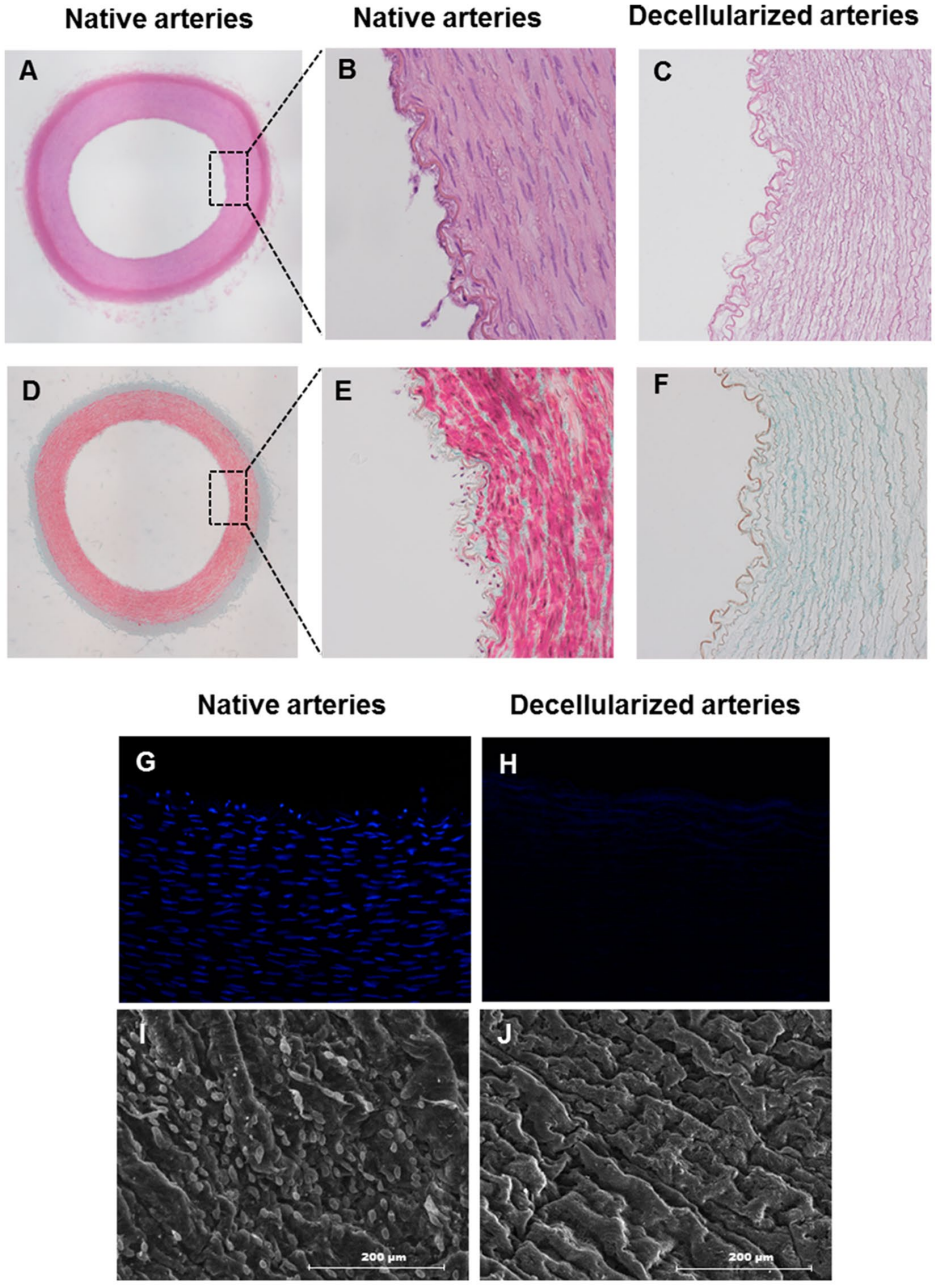
Characterization of porcine carotid arterial tissue before and after decellularization. Histology of porcine arteries: (**A**,**B**,**D** and **E**) native arteries and (**C** and **F**) decellularized arteries. (**A** and **B**) H&E staining of native arteries (magnification; x4 and x10, respectively). (**C**) H&E staining of decellularized arteries demonstrating complete removal of cellular components (magnification; x10). (**D** and **E**) Masson’s Trichrome staining of native arteries showing high cell density embedded in collagens ECM (green fibers) (magnification; 4x and 10x, respectively). (**F**) Masson’s Trichrome staining of decellularized artery revealing preservation of the collagenous ECM and no cells (magnification; x10). Representative fluorescent microscope images of a circumferential cross section stained with DAPI: (**G**) Native arteries and (**H**) decellularized arteries (magnification; x20). SEM micrographs of the luminal surface: (**I**) native arteries and (**J**) decellularized arteries, scale bar indicates 200 μm.

Histological analyses were performed to demonstrate the efficacy of the decellularization process in native carotid arteries. H&E staining showed complete removal of cellular components after the decellularization procedure (Fig. 3C) when compared with native arteries (Fig. 3A,B). In addition, the ECM of decellularized arteries remained intact with the characteristic concentric layers of elastin and collagen (stained pink) (Fig. 3C). Complete removal of vascular cells was confirmed by Masson’s trichrome staining. Native tissue showed high density of﻿ cells embedded in collagen fibers (stained turquoise) (Fig. 3D,E), while no cells were found in the decellularized arteries, which displayed a well preserved ECM of collagen and elastin fibers that maintained the same circumferential orientation of the native tissue (Fig. 3F). DAPI staining was used to further confirm the removal of all cells in the vessel walls. Native arteries stained with DAPI showed cell nuclei at the intimal and medial layer (Fig. 3G), while no nuclei were detected in decellularized arteries (Fig. 3H). SEM analyses of the luminal surface of decellularized arteries confirmed the removal of cellular debris (Fig. 3J). Also, the basic extracellular microstructure of decellularized arteries did not appear altered (Fig. 3J) compared to native ones (Fig. 3I).

### Charaterization of polymer coating and mechanical testing

**Figure 4:**
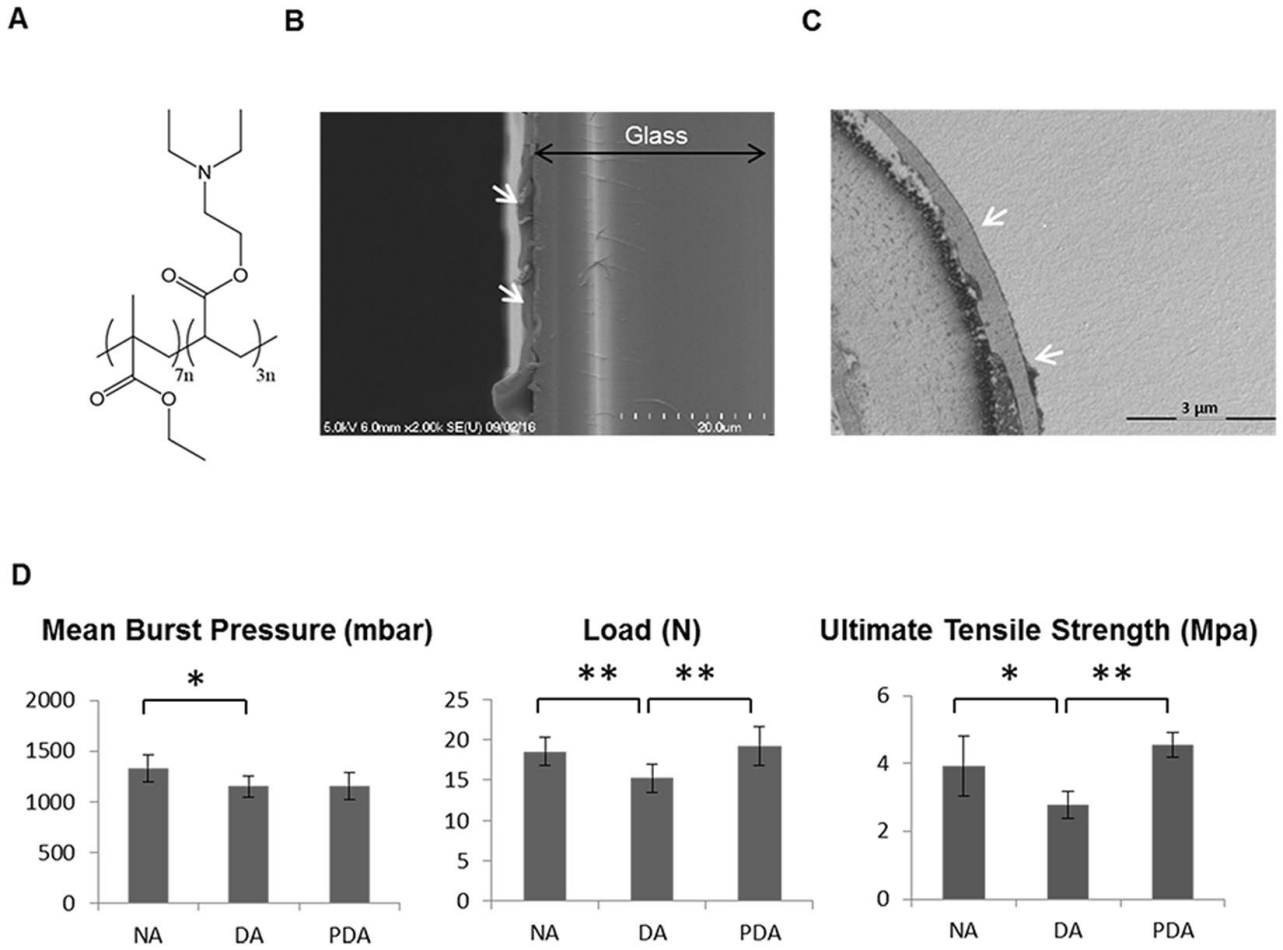
Polymer coating and mechanical test. (**A**) Chemical structure of 8g7 polymer. (**B**) Scanning electron microscopy image of glass coverslip coated with the 8g7 polymer (white arrows). (**C**) Transmission electron microscopy image of polymer-coated decellularized artery section. Polymer coating (white arrows). (**D**) Mechanical analyses of native arteries (NA), decellularized arteries (DA) and polymer-coated decellularized arteries (PDA). Measurements of mean burst pressure, load and ultimate tensile strength are compared between artery groups(*P < 0.05), (**P < 0.01).

The chemical structure of the 8g7 (poly(ethyl methacrylate-co-diethylaminoethyl acrylate)) polymer containing tertiary amine is shown in Fig. 4A. The polymer was coated on glass coverslips and the thickness of the polymer coating was determined by SEM (Fig. 4B) to be 1.78 ± 0.51 μm. In addition, polymer coated decellularized arteries were analyzed by TEM and the thickness of the polymer layer on luminal side was found to be 0.63 ± 0.2 μm (Fig. 4C). The mechanical testing revealed significant differences in burst pressure between the native arteries (1330 ± 135 mbar) and decellularized arteries (1115 ± 108 mbar) with the polymer-coated decellularized arteries (1153 ± 138 mbar) showing marginal improvement (Fig. 4). The polymer-coated arteries were able to withstand a maximum load of 19.2 ± 12.4 N with an ultimate tensile strength for circumferential stress of 4.6 ± 0.4 MPa, which resembled the mechanical properties of native vessels (maximum load = 18.5 ± 1.8 N and ultimate circumferential tensile strength = 3.9 ± 0.9 MPa). However, decellularized arteries showed a significantly lower maximum load (15.2 ± 1.7 N) and ultimate tensile strength (2.8 ± 0.4 MPa) (Fig. 4).

### Biopolymer coated vessels support HUVECs growth

**Figure 5:**
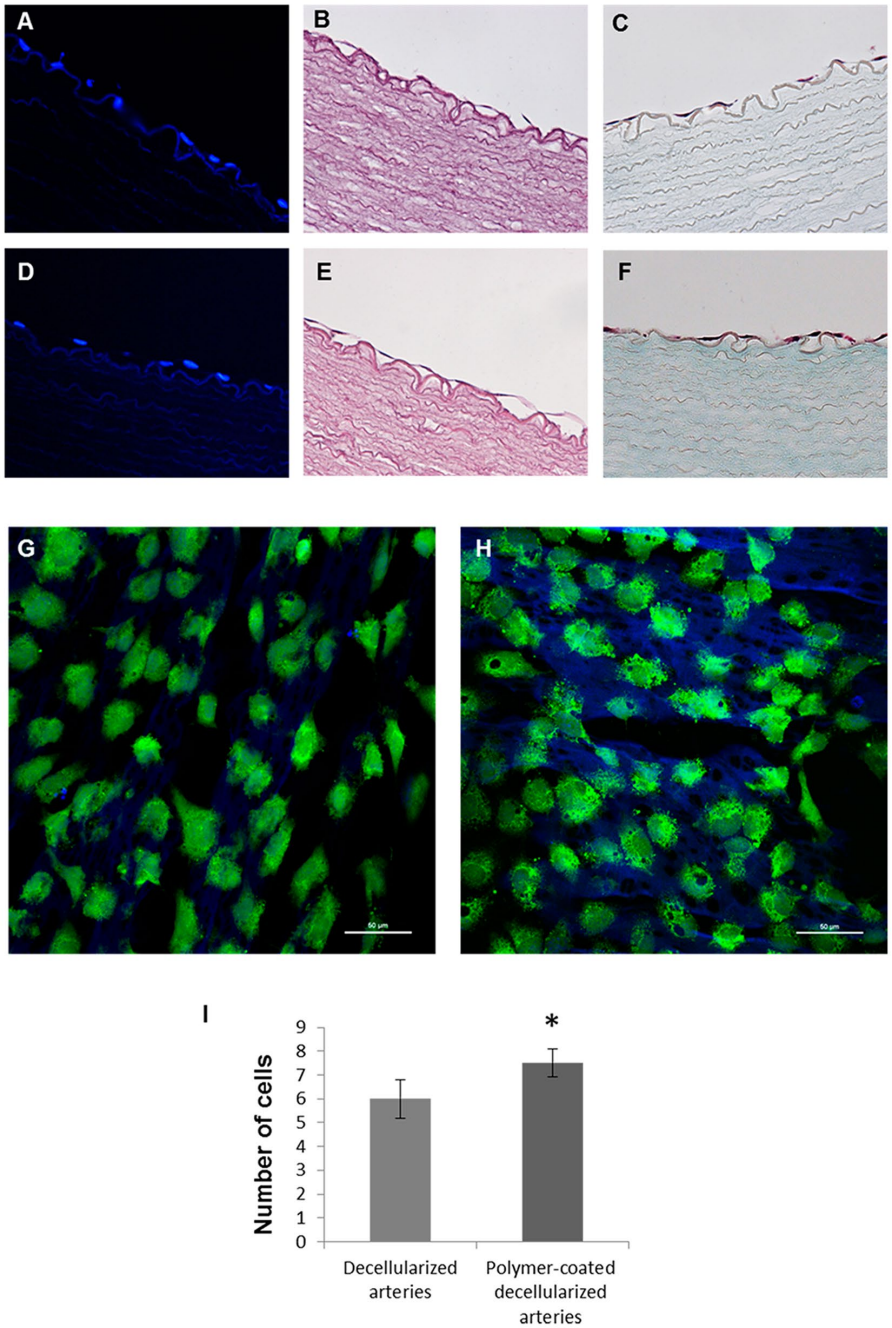
Porcine recellularized arteries after 5 days in culture. Representative fluorescent microscope images of the nuclei stained with DAPI (blue): (**A**) decellularized arteries and (**D**) polymer-coated decellularized arteries (magnification; x40). H&E staining showing endothelial cells: (**B**) decellularized arteries and (**E**) polymer-coated decellularized arteries. Masson's Trichrome staining: (**C**) decellularized arteries and (**F**) polymer-coated decellularized arteries (magnification; x40). Confocal microscope images of live cell labeled with cell tracker green: (**G**) decellularized arteries and (**H**) polymer-coated decellularized arteries. Scale bar = 50 μm (magnification; x40). (**I**) Cell number determination by ImageJ Software on four representative histological staining images of decellularized arteries and polymer-coated decellularized arteries at 40x magnification from independent experiments (*p < 0.05).

We studied the ability of 8g7 to support ECs growth in order to reconstitute the endothelial layer of the decellularized vessels. The coating protocol was optimized to ensure coating of the entire intimal layer of the decellularized vessel using an *ad hoc* system (Figure S2). HUVECs were used to repopulate the endothelium of the decellularized arteries. Cells were seeded over the entire internal layer of decellularized and polymer-coated decellularized arteries and histological analyses were performed to study cell attachment. DAPI staining of vessel cross-sections showed cell nuclei aligned over the lumen surfaces of both uncoated (Fig. 5A) and polymer-coated recellularized arteries (Fig. 5D), indicating that cells attaching to the polymer formed an outline similar to native arteries (Fig. 3G).

Further staining with H&E and Masson’s Trichrome staining confirmed these results (Fig. 5B,C,E,F) and indicated that the biopolymer supported the formation of a more homogeneous interconnected cell layer (Fig. 5E,F).

Confocal microscopy analyses showed a confluent monolayer of ECs in the section of the vessel lumen. Cell tracker green staining evidenced live cells forming a homogenous carpet on the internal surface of both uncoated (Fig. 5G) and polymer-coated recellularized arteries (Fig. 5H). HUVECs adhered tightly to the inner layer of decellularized arteries surface and were able to grow and migrate into the pores created by the removal of the cells after the decellularization process (Fig. 5G,H). The intimal layer of polymer-coated recellularized arteries appeared regenerated with cells arranged following a wave-like alignment of the luminal fibers (Fig. 5H). Moreover, we found a significant increase in the number of cells attached to the polymer-coated decellularized arteries when compared with the decellularized arteries (Fig. 5I).

### Ultrastructural analyses

**Figure 6:**
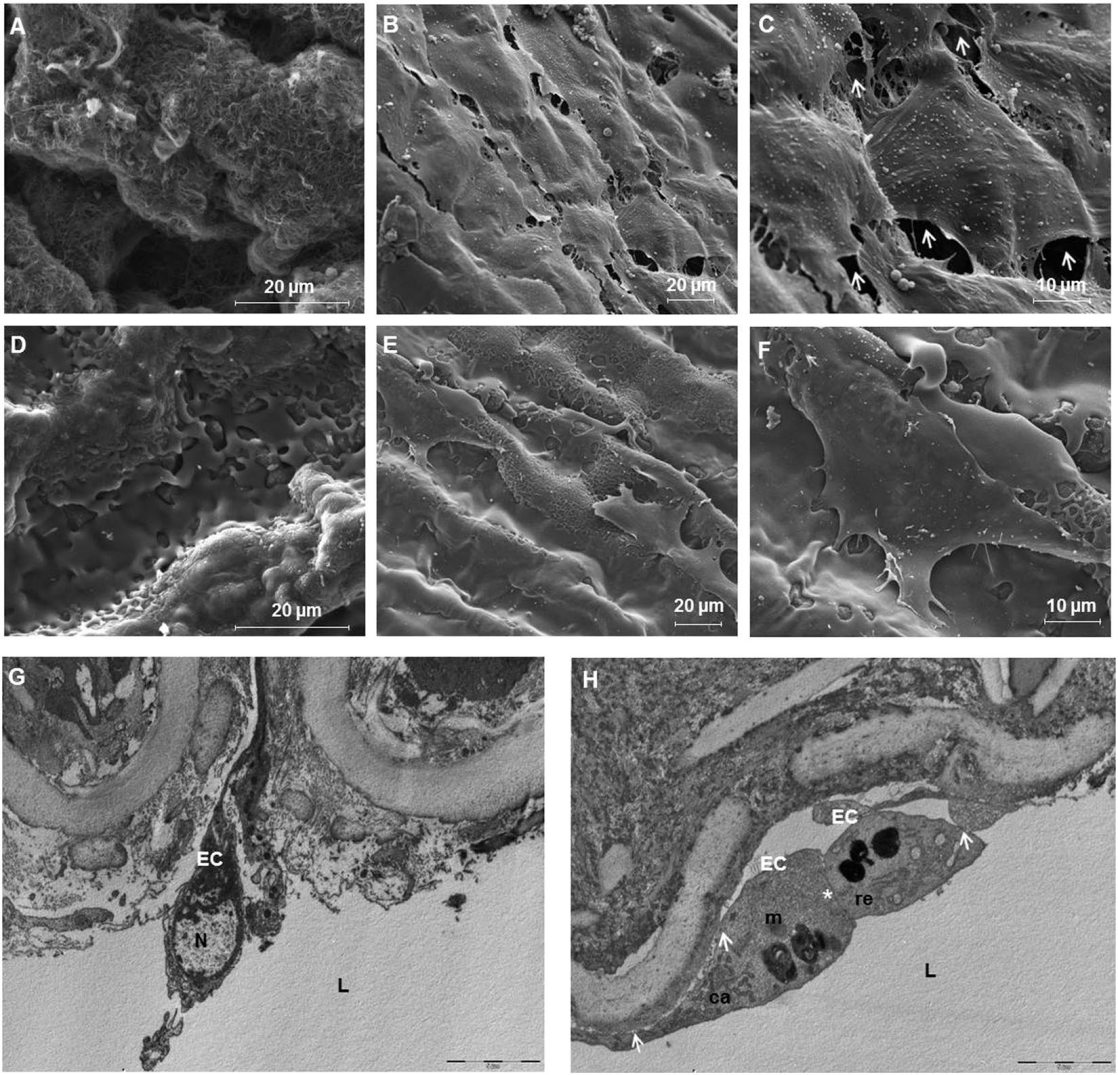
Porcine recellularized arteries after 5 days in culture. Representative scanning electron microscopy (SEM) images of the luminal side of the arteries: (**A**) decellularized artery with no cells, (**B** and **C**) non polymer-coated recellularized arteries, white arrows are pointing out gaps between cells, (**D**) polymer-coated decellularized arteries, (**E** and **F**) polymer-coated recellularized arteries showing the formation of a continuous cell monolayer. (Magnification; (**A**,**C**,**D** and **F**) x4000, (**B** and **E**) x1600). (**G** and **H**) Transmission electron microscopy (TEM) analyses of sections cut in perpendicular to the long axis of the artery of (**G**) native arteries and (**H**) polymer-coated recellularized arteries. (**H**) White asterisk show cell adhesion between two cells and white arrows point out adhesion between the cell and the luminal side of the vessel. L: lumen, EC: endothelial cells, N: nucleus, ca: caveolae, er: rough endoplasmic reticulum, m: mitochondria, scale bar indicates: (**A**) 10 μm, (**B**) 5 μm.

Scanning electron microscopy was performed on artery samples (Fig. 6A–F) with SEM images of a decellularized artery without polymer coating and a polymer-coated decellularized artery showing homogeneous and uniform coating. A suitable endothelial surface was achieved on non polymer-coated (Fig. 6B,C) and polymer-coated (Fig. 6E,F) arteries after recelluarization (5 days culture with HUVECs) with most of the seeded HUVECs distributed on the intima layer, forming a cell mono-layer. The polymer-coated recellularized arteries (Fig. 6E,F) showed a more compact cellular distribution when compared to uncoated ones (Fig. 6B,C). In fact, for the 8g7 coated decellularized arteries, no gap between cells could be found while the uncoated recellularized arteries showed clear gaps between cells (Fig. 6C, white arrows) indicating a discontinuous cell re-endothelialization. These observations demonstrate that 8g7 increases cell attachment and improves cell coverage of the luminal surface while maintaining the cobblestone morphology of the cells.

To confirm these results, TEM analyses of both, native and polymer-coated recellularized arteries sections (cut perpendicular to the arteries) were performed. Micrographs showed the attachment of seeded cells on the intimal surface of the arteries (Fig. 6G,H). Caveolae, a typical feature associated to HUVECs were observed (Fig. 6H). Furthermore, the polymer seemed to facilitate the formation of a continuous epithelium, because specific cell unions, such as cell junctions (white asterisk) and cell adherence to the ECM (white arrows) were observed (Fig. 6H).

## Discussion

In vascular tissue engineering, replacement grafts should recreate the conditions for cellular and tissue organization. Decellularized vessels could provide a promising alternative to synthetic vascular grafts that present disappointing failure rates when replacing small diameter vessels^2, 15^. Moreover, the poor health of patients with vascular diseases restricts the possibility of obtaining homograft tissues. Thus, the use of decellularized xenografts may be a feasible alternative with potential unlimited availability^16^. In fact, xenogeneic ECM has already been used successfully as a scaffold to replace/repair numerous tissues and organs in both preclinical animal studies and human clinical applications^7^. However, the outcomes of the few clinical studies have raised controversies due to poor decellularization, fibrosis and a strong inflammatory response elicited by the xenogeneic collagen matrix^17, 18^. Moreover, it has been shown that the decellularized porcine matrix acts as a platelet-activating surface. Platelet adhesion and aggregate formation was shown to be favored only on the surface of decellularized heart valve matrix or partially denuded matrix whereas after seeding with ECs no platelet activation was detected^19^. Therefore, avoiding the direct exposition of the acellular surface to the blood and a healthy confluent endothelial layer seem to be critical requirements to circumvent adhesion and aggregation of platelets and, thereby, graft rejection and thrombosis. Consequently, our study aimed to develop decellularized artery scaffolds that provide a suitable microenvironment to support ECs expansion while diminishing platelet adherence capabilities.

Many strategies have been developed to repopulate vascular grafts and establish a complete endothelium including among others the coating of decellularized heart vessels with substrates such as fibronectin^20^, heparin and vascular endothelial growth factor (VEGF)^21–24^. However, synthetic polymer coatings are more suitable for clinical applications as they are well defined materials, can be synthesized reproducibly and according to GMP standards. In a previous study polyurethane and polyacrylate libraries were screened using polymer microarray technology^13^, to identify synthetic biopolymers with essential attributes to support adhesion and expansion of both progenitor and mature ECs while minimizing platelet attachment. The best selected polymer (8g7) demonstrated comparable levels of binding for both EPC and mature ECs and an equivalent high expression of endothelial surface markers when compared to long-established substrates for *in vitro* culture conditions i.e., collagen type I and polystyrene^13^.

Based on that previous work, this study was designed to combine the properties of ECM-based natural grafts and 8g7 biopolymer to generate a tissue-engineered vascular scaffold that supports re-endothelialization and reduced platelet aggregation. First, the adhesion, growth and capillary tube formation abilities of ECs seeded on 8g7 were demonstrated. ECs grown on polymer-coated coverslips showed their functional capacity to induce endothelial tube-like structures similar to matrigel. Despite extensive use of matrigel for the attachment and differentiation of ECs, based on its ability to provide a suitable microenvironment to promote angiogenesis and endothelial proliferation^25^, it is derived from mouse sarcomas and hence unsuitable for clinical applications due to the risk of pathogen transmission and immunogenicity. From a clinical point of view, the growth of ECs needs a fully defined microenvironment which could be provided by chemically defined synthetic polymers such as 8g7. We hypothesize that a balance between the mechanical properties of the polymer and the growth factors of the culture medium may regulate the formation of tubule-like structures. In fact, the characteristics of the substrate have been shown before to direct cytoskeleton organization and to affect cell adhesion and disposition^26^. Other factors such as the substrate stiffness have been shown to influence the formation of tubule-like structures^27^ with softer substrates showing better capillary network, indicating that the mechanical characteristic of the substrate have an impact on endothelisation^28, 29^.

Previous studies have demonstrated the efficacy of other substrates to reduce platelet attachment^30^, although results are interesting, it is relevant to note that those studies were performed under static parameters. Here, we established the ability of 8g7 to reduce platelet aggregation under flow conditions using a dynamic method that mimics physiological flow. As platelet adhesion and aggregation mechanisms are shear stress dependent^31^, *in vitro* flow devices, such as parallel-plate flow chambers that mimic the *in vivo* conditions, are widely used for the analysis of platelet function in whole blood over the range of physiological shear stresses^32^. Such devices can also be used to evaluate thrombogenic potential of platelet over coated surfaces^33, 34^. Interestingly, perfusion of whole blood in a parallel-plate flow chamber demonstrated that 8g7-coated coverslips exhibit reduced platelet adhesion and aggregation in increasing shear rates in comparison to the uncoated coverslips. This significant decrease in platelet attachment confirms the anti-thrombotic properties of 8g7 under a pseudo-physiological flow. The adhesion of cells to a biomaterial surface has been proposed to be a two-step process, in which the first step of ‘passive adhesion’ is controlled by physicochemical interactions (such as hydrophobicity and surface charge) and the second step of ‘active adhesion’ achieved due to the interactions of surface proteins on the cell and substrate^35, 29^. Though physicochemical interactions such as hydrophilicity can play an important role in cellular attachment, as seen in temperature-modulated poly(N-isopropyl acrylamide) gels, the detachment temperature (and hence the level of hydrophilicity) is known to be dependent on the cell type (e.g., hepatocytes or endothelial cells)^35^, thus highlighting a much more complex association between cell binding and substrate hydration. Similarly, the influence of electrostatic charges on cell-binding is not fully understood. Moreover, different proteins have varying affinity to a polymer surface; and the interaction of proteins attached to the substrate and the receptor proteins on the cell surface has an important role in cell adhesion. It has been shown that platelet adhesion is not only related to the hydrophilicity of the substrate and the amount of plasma proteins absorbed but also on their conformation. Hence two surfaces poly(hydroxyethyl methacrylate) and poly(2-methoxyethylacrylate), both allow low protein binding, however, poly(2-methoxyethyl acrylate) that preserves the native conformation of adsorbed proteins displays remarkably lower platelet binding^36^.

Among the several protocols described for decellularization of vessels, various studies have shown that enzyme-based decellularization techniques lead to the destruction of the ECM^37^. However, in the protocol used in this study, previously reported by Seridan *et al.* ^38^, trypsin treatment was applied for a short period of time followed by treatment with Triton X-100, which effectively removed the cellular material leaving an intact ECM structure formed mainly by collagen and elastin proteins as shown by histological and SEM analyses. These results are also similar to other studies which used Triton X-100 as a detergent for artery decellularization and showed a well preserved ECM^39, 40^. Though the decellularization procedure did not cause extensive damage to the extracellular matrix of the arteries, it resulted in poor mechanical properties, in agreement with previous studies^41, 42^. In the current work, mechanical tests revealed that uncoated decellularized arteries ruptured at a lower pressure than native arteries, as previously reported^43^ and coating with the 8g7 polymer enhanced the mechanical properties of the decellularized vessels. Combination of decellularized vessels with polymers such as poly ε-caprolactone (PCL)^44^ and poly(D,L-lactide) (PDLLA)^45^ has been reported to improve the biomechanical properties of the graft. Due to their diverse mechanical properties, a wide range of biocompatible polyacrylates has found varied applications in biomedicine ranging from poly(methyl methacrylate) (PMMA) used in artificial teeth and bone cements to polyhydroxyethyl methacrylate used in soft contact lenses, drug delivery systems and dressings^46–50^. Vascular grafts made of polyacrylates are also commercially available^51^. In this work we have demonstrated that the mechanical properties of decellularised arteries improved significantly when coated with poly(ethyl methacrylate-co-diethylaminoethyl acrylate). In general, poly(ethyl methacrylate) based polymers have Young's modulus lower than ‘hard’ polyacrylates (such as PMMA) and higher than ‘soft’ polyacrylates (such as poly(ethyleneglycol methacrylate)^52^.

In spite of the excellent physical and mechanical properties of promising synthetic polymers like poly(vinyl alcohol), *in vitro* and *in vivo* studies have failed to show any significant endothelial cell attachment on the grafts luminal surfaces^53^. Moreover, *in vitro* experiments with grafts made of synthetic materials currently in clinical use showed that ECs produced isolated clumps and appeared as small spheroids with single point contact^54^. Synthetic vascular grafts coated with either antibodies or integrin-binding peptides have been developed for increasing cell adhesion to the inner lumen directly from the circulating blood stream^12, 55–57^. However, nonspecific adhesion of non-EPC remains major concern as this can lead to problems such as restenosis, inflammation or hyperplasia^58^. Thus, physical and biochemical surface modifications have been seen to be necessary to improve the cell attachment on these synthetic polymers^53, 59, 60^. From a clinical point of view, the growth of ECs needs a fully defined microenvironment which could be provided by chemically defined synthetic polymers such as 8g7.

Polymer 8g7 provides a superior substrate for attachment and proliferation of ECs where cells appeared homogenously distributed and exhibit the typical cobblestone monolayer morphology associated with high levels of attachment and enhanced cellular proliferation^54^. Moreover, decellularized tissue-engineered arteries can be stored and thus made readily available for patients similar to synthetic graft materials. Emerging approaches have started to engineer coated grafts by combining biological tissue and synthetic polymers. Recently, Gong *et al*. fabricated a hybrid tissue engineered vascular graft by the application of an electrospun nano-polycaprolactone coating on the outside of a decellularized rat aorta, which improved the biomechanics of the decellularized vessel. However, they also needed to incorporate exogenous heparin to modify the acellular vascular intimal surfaces^44^. In another study, the anticoagulant lepirudin was added to a Poly-(D,L-lactide) coating applied in decellularized arteries. Although it showed a reduction in the thrombogenicity and in the ECM’s rate of degeneration, no evidence of recellularization was documented after *in vivo* implantation of the graft, suggesting that polymer coating might result in inhibiting cellular repopulation^45^.

Here, we demonstrated for the first time that the endothelium of decellularized arteries is regenerated by coating with a synthetic polymer with lower affinity for platelets while showing improved mechanical properties.

These results suggest that polymer coating of decellularized arteries may improve the *in vivo* blood compatibility of decellularized vessels and encourage future implantation studies to evaluate its patency, resistance to haemodynamic forces preventing thromboembolic events necessary for the long-term performance of small diameter vessels.

## Conclusions

Our study demonstrates a novel method of coating decellularized vessels with poly(ethylmethacrylate-co-diethylaminoethyl acrylate). This material is biocompatible and readily synthesized. The polymer coating enhances attachment of ECs promoting endothelium regeneration, while it is able to reduce platelet attachment and improve the mechanical properties of decellularized vessels. This work suggests that the combination of the natural vascular architecture of decellularized arteries with the properties of 8g7 appear to be ideal for the development of chimeric tissue engineered vessels aiming to promote endothelialization and prevent thrombosis. These novel mixed grafts could help to overcome the current compliance mismatch between synthetic grafts and natural arteries.

## Materials and Methods

### Polymer synthesis

All chemicals were purchased from Sigma Aldrich (Gillingham, UK) and used as supplied without purification. Protocols are detailed in the supplementary section (Tables S1 and S2 and Figure S1). All methods and experimental protocols were approved and carried out in accordance to the guidelines and regulations of the University of Granada.

### Polymer coating of coverslips

Polymers were spin-coated onto glass coverslips. Two sizes of glass coverslips were used, circular (19 mm diameter) and square (18 × 18 mm) (Menzel-Gläser, Braunschweig, Germany). Polymer solutions in THF (2% w/v) were spin-coated at 2000 rpm for 10 seconds using a spin coater (6708D, Speedline Technologies). The coated surfaces were dried in a convection oven at 40 °C overnight and sterilized using UV light prior to cell culture.

### Capillary-like tube formation assay on polymer-coated coverslips and matrigel

HUVEC cell line was obtained from American Type Culture Collection (ATCC® CRL-1730™) and cultured following ATCC recommendations. HUVECs were cultured in complete EGM-2 growth medium (Lonza, Basel, Switzerland) under standard cell culture conditions of 37 °C and 5% CO_2_. Cells were used between passages 4 and 5. Endothelial cells grown on matrices such as matrigel are able to migrate and align forming capillary-like or cord-like structures^61, 62^. Here we have assessed if the 8g7 polymer can mimic matrigel’s properties and induced the HUVECs cells to form the described tubule-like structures. To do so, HUVECs were seeded and incubated on polymer coated and uncoated coverslips (30000 cells/well) at 37 °C and 5% CO_2_ in 12-well plates. In parallel, HUVECs were cultured in matrigel (BD Biosciences, San Jose, CA) coated 12-well plates as a control. After 4 h, cells were imaged (Nikon Eclipse Ti-U, USA) and the number of capillary-like structures was measured. Each cord portion between the ramifications was considered as one capillary unit^14, 61^. Mean ± standard deviation (SD) values were obtained by evaluating four representative areas of each culture from three independent experiments and carried out by two observers. Additionally, the average number of cells that form each branch of the tubule-like and the mean number of branching points (the nodes where branches meet^63^) per each capillary-like structure formed were quantified from four random fields at 10x magnification and obtained from three independent experiments using ImageJ^﻿™^ program^62^.

### Platelet adhesion on the polymer under flow conditions

Platelet adhesion on uncoated and polymer-coated coverslips was assessed using a parallel-plate perfusion chamber^64, 65^. Blood from healthy human donors was drawn by venipuncture and processed immediately after collection^13^. Informed patient consent was obtained for all samples used in this study and samples were collected in accordance with the Research Ethics Committees of the Hospital Clínic of Barcelona (CEIC 2009/4700), and University of Granada. Two coverslips were individually inserted into the separate receptacles of the perfusion chamber and citrate-anticoagulated blood was recirculated at shear rates of 600 s^−1^, 800 s^−1^, and 1200 s^−1^ for 5 min. The coverslips were then rinsed with 0.15 M phosphate buffered saline (PBS), fixed with 0.5% glutaraldehyde in 0.15 M PBS at 4 °C for 24 h and stained with 0.02% toluidine blue for morphometric evaluation. The perfused surface was imaged using a video camera (Leica) fitted to a light microscope (Polyvar, Reichert-Jung). Two independent experiment were performed (n = 2) and percentage of the area covered by platelets of 3 different areas per coverslip was obtained using ImageJ™ program, by comparing the area of platelet coverage to the area of the image.

### Tissue harvest

Porcine carotid arteries were obtained from a slaughterhouse and transported to the laboratory stored in cold PBS with 1% penicillin/streptomycin. Immediately after arrival, carotid arteries were cleaned of excess of connective and adventitial tissues and rinsed in sterile PBS. Common carotid arteries 3–4 mm in diameter were cut into approximately 3–5 cm long segments (Figure S2), frozen in PBS at −80 °C and defrosted just before use.

### Decellularization process

The decellularization process of carotid tissue samples was performed as previously described using enzymatic digestion and detergent extraction^38^. Carotid arteries were immersed in de-ionized water for 24 h at 4 °C followed by treatment with 0.05% Trypsin with 0.02% EDTA (Sigma Aldrich, St. Louis, MO, USA) for 1 h at 37 °C. After a short rinse in PBS to remove excess trypsin, the samples were treated with a solution of 2% Triton X-100 and 0.8% ammonium hydroxide (Sigma) in de-ionized water for 72 h at 4 °C changing the solution every 24 h. After the decellularization process, samples were washed in de-ionized water three times, for 24 h each, to remove chemical residues. All steps were carried out under continuous shaking except trypsin incubation.

### Polymer-coating of decellularized arteries

After cannulation with a three-way stopcock, (see Supplementary Data, Figure S2), 300 μL of the 8g7 polymer solution (2.0% w/v in 25% acetic acid) was injected into the decellularized arteries and incubated for 30 min at 4 °C under gentle shaking. Decellularized arteries were also injected with 25% acetic acid solution as a control. Coated and uncoated decellularized carotid arteries were dried 2h at room temperature in a fume hood, washed twice with PBS and irradiated with UV light for 30 min before use.

### Mechanical testing

Samples were divided into three groups: (i) native arteries, (ii) decellularized arteries and (iii) polymer-coated decellularized arteries. The biomechanical properties of all the groups (n = 3, in each group) were determined by conducting uniaxial tensile tests using an *ad hoc* and lab-made device (Figure S3). The controlled static load by pumping water (using a peristaltic pump) at 8 grams increments (with a pause of 20 seconds after each increment), while keeping the vessels continuously hydrated. The strain was measured by image correlation techniques from a high-resolution video. The video was triggered at the beginning of pumping and a custom image capture software was used to image at a rate of 1 frame/increment. This allowed to track the motion of the speckle pattern applied on the tissue, and their large displacements were calculated by analyzing the recorded images (Figure S4) as previously reported^38, 66^.

Ultimate tensile strength, load and burst pressure were determined. For ultimate tensile strength and load, ring sections were cut from each sample, mounted on parallel aligned custom-made holders and loaded until failure. The burst pressure was calculated from their failure load where radial stress was calculated and converted to millibars taking into account load, mean radius and ultimate circumferential tensile strength^67^. Diameter, width, thickness and length of each sample used for the calculations were measured using a high-precision slide caliper.

### Arteries recellularization

Both, 8g7 polymer coated and uncoated decellularized carotid arteries were pre-incubated overnight in complete EGM-2 medium (Lonza, Basel, Switzerland) cannulated with a three-way stopcock. The excess media was removed and HUVECs (at passage 4, 1 × 10^6^ cells/mL) were seeded onto the luminal surface of the arteries (length, 15 mm; inner diameter, 4 mm) (n = 3). Vessels were rotated 90° around its longitudinal axis every 30 min until all surfaces had been exposed to cells. After 360° rotation (2 h), cannulated arteries were immersed in media and the graft was incubated for another 24 h to ensure complete cells attachment. After 24 h and every 2 days thereafter, medium was changed. Non-seeded processed arteries were maintained in media and served as control samples. Cells were maintained in culture for 5 days before being collected for analysis.

### Histological and immunofluorescence analyses

Samples were divided into five groups: (i) native arteries, (ii) decellularized arteries, (iii) polymer-coated decellularized arteries, (iv) non polymer-coated recellularized arteries and (v) polymer-coated recellularized arteries. Ring segments of 5 mm thickness were taken from each sample group for histological analyses. Samples were fixed in 4% paraformaldehyde, embedded in paraffin in an automatic tissue processor (TP1020, Leica, Germany), cut into 5 μm sections and stained with Hematoxylin and Eosin (H&E) and Masson's Trichrome as previously reported^68^. The number of cells on decellularized arteries and polymer-coated decellularized was quantified from four representative histological staining images at 40x magnification from three independent experiments using ImageJ™ program. For 4′, 6-diamidino-2-phenylindole (DAPI, Sigma) staining, slides were deparaffinized, rehydrated and stained with mounting medium (Vectashield, Burlingame, CA, USA) containing DAPI. Bright field and fluorescent (DAPI) images were acquired with an inverted microscope (Nikon H550s, USA).

### Laser confocal microscopy

Samples of uncoated and polymer-coated recellularized arteries were washed twice with PBS and labeled with the fluorescent dye, CellTracker Green/CMFDA (Invitrogen Inc., Carlsbad, CA, USA) following the manufacturer’s instructions. Arteries were fixed with 4% paraformaldehyde in PBS for 20 min at RT and stained with DAPI (1 μg/mL) in PBS. Cells on the luminal surface were imaged by confocal microscopy (Nikon Eclipse Ti-E A1, Amsterdam, Netherlands) and analyzed using NIS-Elements software (Amsterdam, Netherlands).

### Scanning electron microscopy (SEM)

Arteries were washed twice with 0.1 M cacodylate buffer (Sigma) and fixed with 2.5% (w/v) glutaraldehyde in 0.1 M cacodylate buffer (pH 7.4; Polysciences, Warrington, PA) for 4 h at 4 °C. Thereafter, samples were rinsed several times with sodium cacodylate buffer. After fixation, samples were post fixed with 1% w/v osmium tetroxide for 1 h RT, dehydrated stepwise with ethanol (50%, 70%, 90% and 100%, 15 min each), critical point dried in CO_2_ and gold coated by sputtering. Images were examined by a FEI Quanta 400 scanning electron microscope (Oregon, USA).

In order to characterize polymer coating on glass, glass coverslips (19 mm) were coated with the 8g7 polymer, following which they were cut, coated with a gold/palladium (60/40%) alloy and imaged along their thickness using a Hitachi 4700 II, cold field-emission Scanning Electron Microscope. The image was processed using ImajeJ program.

### Transmission electron microscopy (TEM)

Samples from native and polymer-coated recellularized arteries were assayed by TEM as previously reported^69^. Briefly, samples were fixed with 2.5% (w/v) glutaraldehyde in 0.1 M cacodylate buffer for 4 h at 4 °C and rinsed several times with 0.1 M cacodylate buffer. Samples were post-fixed with 1% osmium tetroxide for 2 h, washed with 0.1 M cacodylate buffer and dehydrated stepwise in ethanol concentrations (40%, 50%, 70%, 80%, 90%, 95%, 20 min each and two steps in 100%, 15 min each). Samples were then treated with propylene oxide followed by 1:1 propylene oxide and epoxy resin for 3 hours in agitation in a fume hood and then transferred to 100% epoxy resin overnight. Subsequently, they were embedded in fresh epoxy resin and ultra-thin sections (70 nm) were prepared using a diamond knife (Diatome) and contrasted with uranyl acetate and lead citrate prior to examination with a LEO 906E transmission electron microscope (Zeiss, Germany).

### Statistical analysis

All experiments were performed in triplicate. Results are presented as mean ± SD. A Student’s T-test was used to test significance between specific cases. Significant differences for the % surface coverage area of platelets were estimated using the Mann–Whitney U-test. Results were considered significantly different at p < 0.05(*) and p < 0.01(**).

## Electronic supplementary material

Supplementary info
