# Supplementary material for: Poly(ethylmethacrylate-co-diethylaminoethyl acrylate) coating improves endothelial re-population, bio-mechanical and anti-thrombogenic properties of decellularized carotid arteries for blood vessel replacement
Source: Sci Rep. 2017 Mar 24;7:407. doi: 10.1038/s41598-017-00294-6 (PMC5412652; doi:10.1038/s41598-017-00294-6)
Supplement: Supplementary file 1 — Supplementary info [file 41598_2017_294_MOESM1_ESM.docx]

**Poly (ethylmethacrylate-co-diethylaminoethyl acrylate) coating improves endothelial re-population, bio-mechanical and anti-thrombogenic properties of decellularized carotid arteries for blood vessel replacement.**

E López-Ruiz, S Venkateswaran, M Perán, G Jiménez, S Pernagallo, JJ Díaz-Mochón, O Tura-Ceide, F Arrebola, J Melchor, J Soto, G Rus, PJ Real, M Diaz-Ricart, A Conde-González, M Bradley, JA Marchal.

***Polymer synthesis and characterization***

The polymer 8g7 was synthesized by free-radical polymerization, using 2,2’-azobis (2-methylpropionitrile) (AIBN) as an initiator. The monomers, AIBN and the solvent were added to a glass vessel, and polymerization carried out for 48 h under N_2_ atm. Reaction conditions are summarised in Table S1. The polymer w precipitated by dropwise addition into hexane, collected by centrifugation, washed with hexane, and dried overnight *in vacuo* at 40 °C. The polymer was characterized by GPC (Table S2) and IR (Fig. S1). GPC was conducted with DMF as eluent (1 mL/min), using an Agilent GPC, fitted with a PLgel 5 µm MIXED-C column (300 × 7.5 mm) at 60 °C, pre-calibrated using polystyrene standards. IR analysis was conducted using a Brucker Tensor 27 spectrometer.

**Table S1:** Reaction conditions - synthesis of 8g7

| **Polymer** | **Monomers (mmol)** | | **AIBN** (mmol) | **Solvent** | **T** (ºC) | **Yield** (%) |
| --- | --- | --- | --- | --- | --- | --- |
| **8g7** | EMA = 26.6 | DEAEA= 11.4 | 0.05 | Toluene  (5.4 mL) | 60 | 67% |

EMA = Ethyl methacrylate*;* DEAEMA = 2-(Diethylamino) ethyl acrylate

**Table S2.** Molecular weight (Mw and Mn) and polydispersity index (PDI) of polymer 8g7.

| **Polymer** | **M_w_** | **M_n_** | **PDI** |
| --- | --- | --- | --- |
| **8g7** | 193000 | 443000 | 2.3 |

**
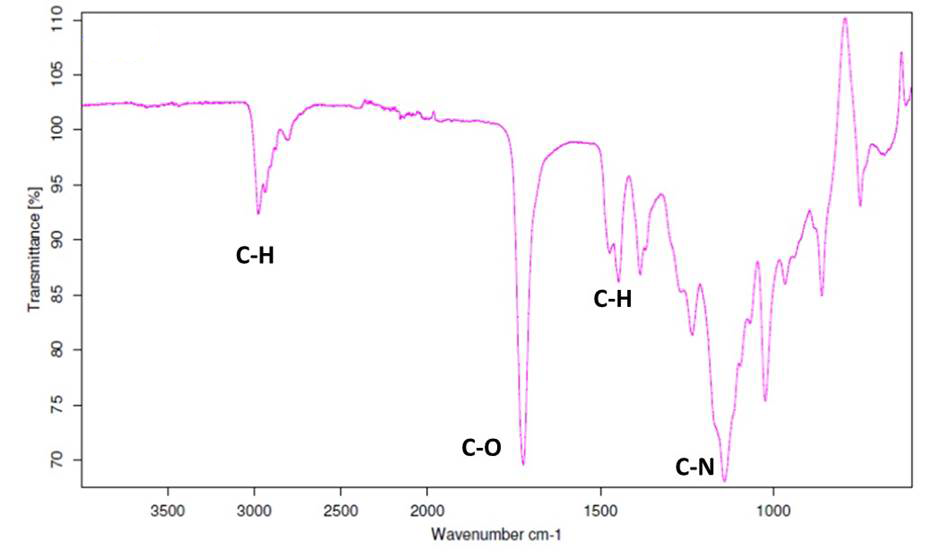
**

**Figure. S1** FT-IR spectra of 8g7 polymer

**
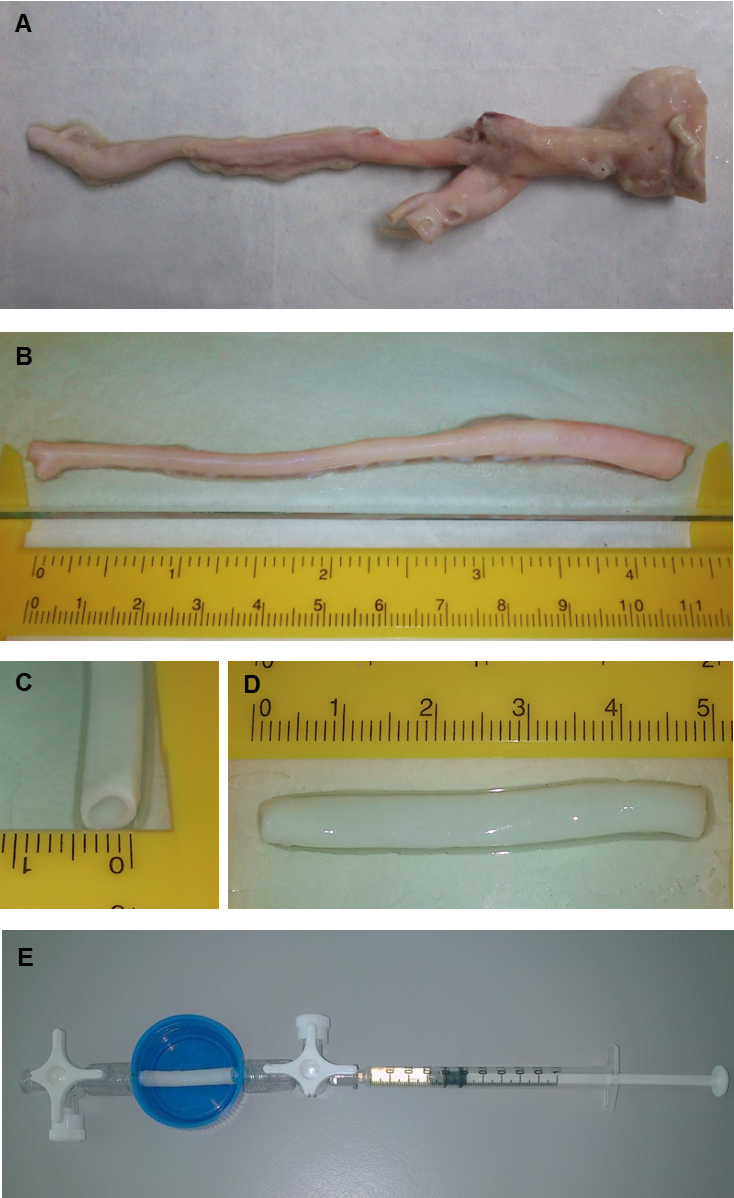
**

**Figure. S2.** Macroscopic images of the left common porcine carotid artery: (A) before removing excess connective and adventitial tissue and (B) after removing excess connective and adventitial tissue and (C and D) after decellularization (artery length ~ 50 mm, inner diameter ~ 4 mm). (E) Cannulated decellularized artery with a three-way stopcock before polymer coating.


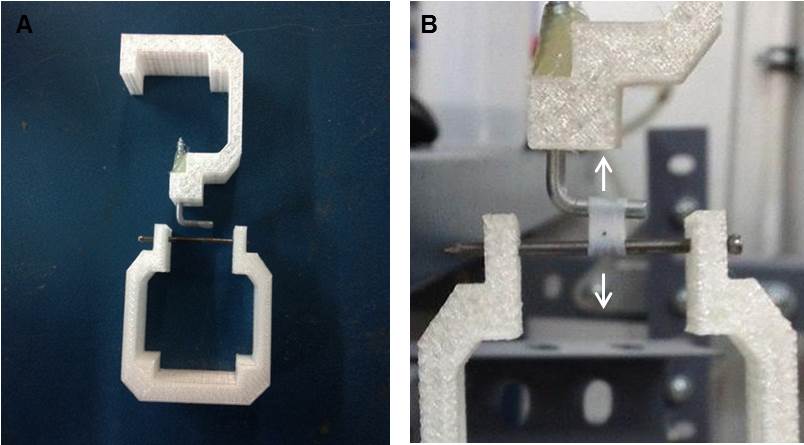


**Figure. S3.** Device fabricated for the tensile and load test. (A) Image of 3D-printed holders. (B) Artery ring sections were mounted on parallel aligned holders and loaded until failure. White arrows indicate the direction of extension.


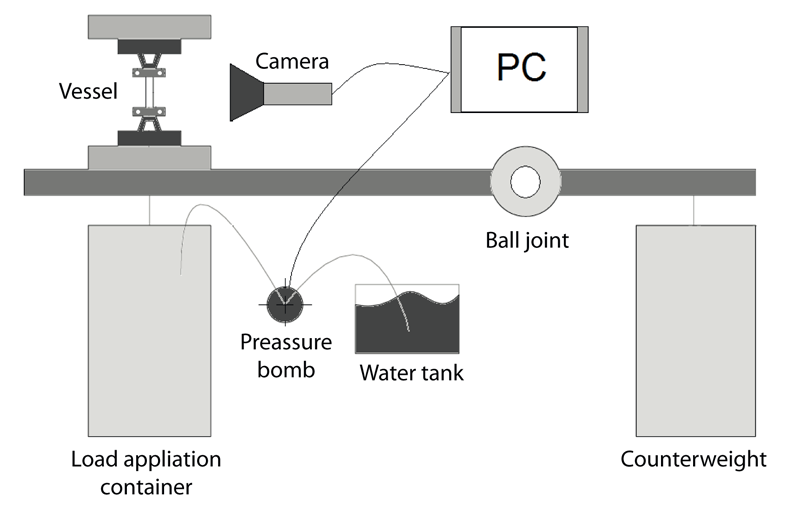


**Figure. S4.** Schematic diagram illustrating the mechanical testing system.
